# Tumor Size as a Critical Prognostic Factor in T1-2 Stage Esophageal Cancer

**DOI:** 10.1155/2020/2796943

**Published:** 2020-08-17

**Authors:** Zilong Wu, Bentong Yu

**Affiliations:** ^1^Department of Thoracic Surgery, The First Affiliated Hospital of Nanchang University, Nanchang, Jiangxi, China; ^2^The First Clinical Medical College, Nanchang University, Nanchang, Jiangxi, China

## Abstract

**Background:**

Tumor size has been measured in esophageal cancer for decades, but the role of tumor size in relation to T stage in the prediction of survival is still underappreciated. Thus, the present study is aimed at investigating the influence of T stage on the predictive value of tumor size in clinical stage I–IV esophageal cancer patients.

**Materials and Methods:**

Data were obtained from the Surveillance, Epidemiology, and End Results Program (SEER) cancer registry program. Cox proportional hazards regression was utilized to identify the independent prognostic ability of the factor. Kaplan-Meier analysis was used to estimate the distribution of survival outcome. Harrell's concordance index (c-index) was used to quantify the predictive ability of the prognostic model and prognostic factor.

**Results:**

According to the T stage, subgroup analysis showed that tumor size was not an independent risk factor in T3 and T4 stage esophageal cancer patients. Furthermore, the predictive power of tumor size was negatively impacted by the increase in T stage. Furthermore, the discriminative ability of the Cox model based on the tumor-node-metastasis (TNM) system with tumor size outperformed the model based on the TNM system only.

**Conclusion:**

The current study identified tumor size as a critical clinical prognostic signature for esophageal cancer with considerable discriminatory ability and prognostic value. Therefore, tumor size should be included in the American Joint Committee on Cancer (AJCC) TNM staging of T1-2 esophagus cancer patients.

## 1. Introduction

Esophageal cancer is the ninth most common cancer type worldwide and the sixth most common cause of cancer death globally [1]. Although the prognosis and survival have improved as result of recent developments in the field, population-based research has shown an overall 5-year survival rate of only 20% [2]. At present, the American Joint Committee on Cancer (AJCC) tumor-node-metastasis (TNM) staging system is widely applied to the diagnosis and treatment of esophageal cancer [3]. However, the clinical outcome differs greatly even among patients with the same TNM stage of esophageal cancer [4]. Therefore, it is important to obtain additional prognostic factors to improve prognosis prediction and to further classify the stages.

The AJCC TNM staging system is the most widely used clinical prognostic classification system and classifies patients by the primary tumor (T), regional lymph nodes (N), and distant metastasis (M). The T category for clinical stage I–IV esophageal cancer informs on the depth of tumor invasion and includes T1–T4 [3].

Tumor size is defined as the maximum length of the tumor and is widely used in the AJCC T staging system in many types of cancer, including lung cancer and breast cancer [5, 6]. As an easily acquired clinical prognostic factor, research has shown that larger tumors indicate worse prognosis in esophageal cancer [7–11], and T stage is a powerful prognostic factor in esophageal cancer. Some researchers have explored the value of tumor size on prognosis in each T classification [12–15]. Nevertheless, a previous study has explored the influence of T stage on the predictive ability and prognostic effect of tumor size in esophageal cancer. As an important clinical prognostic factor, the size of the tumor may unequally work in a different subgroup of esophageal cancer based on infiltrating depth. In the current study, we performed a retrospective analysis of the Surveillance, Epidemiology, and End Results (SEER) database (a registered database), in order to explore the influence of T stage on the predictive ability and prognostic effect of tumor size in esophageal cancer.

## 2. Method

### 2.1. Data Resource

Data were downloaded from the SEER database (https://seer.cancer.gov/), 18 population-based cancer registries, by the SEER ∗ Stat (version 8.3.6). The SEER database is a cancer registry with excellent data quality and near-complete case ascertainment [16]. The SEER database includes data on approximately 30% of the U.S. population, which is publicly available and deidentified; thus, this study was exempt from local institutional review board review.

### 2.2. Patient Selection

In total, 37161 esophageal cancer patients who were diagnosed between 2004 and 2015 were included in the research. The inclusion criteria were as follows: (1) esophageal cancer was diagnosed by pathology; (2) histological types were limited to squamous cell neoplasms (8050–8089), adenomas and adenocarcinomas (8140–8389), epithelial neoplasms (8010–8049), cystic, mucinous, and serous neoplasms (8440–8499), complex epithelial neoplasms (8560–8579); and (3) esophageal cancer was the only primary tumor. Cases with missing or unclear information, such as that relating to follow up and primary tumor size, were excluded from the study. Ultimately, 17845 patients were included in this research from the SEER database.

### 2.3. Covariates and Outcome Measures

The following demographic and clinicopathological variables were obtained from the SEER database: age (years); sex (female, male); histology (squamous cell neoplasms (8050-8089); adenomas and adenocarcinomas (8140-8389); epithelial neoplasms (8010-8049); cystic, mucinous, and serous neoplasms (8440-8499); complex epithelial neoplasms (8560-8579); grade (Grade I, Grade II, Grade III-IV, and Unknown); M stage (M0, M1, and MX); N stage (N0, N1, N2, N3, and NX); lymph nodes examined (0-2, >2); tumor size (cm); surgery therapy regimens (no surgery, local tumor excision or/with destruction (local tumor ED), esophagectomy with laryngectomy, and/or gastrectomy(esophagectomy LG)), and follow-up for survival (survival months, vital status, and cause of death). All included patients were restaged according to the eighth AJCC Cancer Staging Manual. Age and tumor size were analyzed as continuous variables in the Cox proportional hazards regression analysis, and the remaining factors were analyzed as categorical variables. The study endpoint was cancer-specific survival (CSS). To reduce the occasionality of the researcher, we also performed the analysis with the endpoint as overall survival (OS). CSS was defined as the duration from the date of diagnosis until death due to esophageal cancer, and OS represented the length of time from either the date of diagnosis or the start of treatment.

### 2.4. Statistical Analysis

R (version 3.5.2, https://cran.r-project.org/bin/windows/base/old/3.5.2/) was used to perform all statistical analyses. Descriptive statistics are presented as the median and percentage. Using X-Tile, some continuous variables were transformed into categorical variables [17]. Univariate and multivariate Cox proportional hazards regression analyses were performed to confirm the independent prognostic role of the factors. The Kaplan-Meier method was used to estimate the distribution of survival outcome according to the group. Harrell's concordance index (c-index) was used to assess the ability of prognostic factors and prognostic models to predict survival [18]. Greater c-index of a prognostic factor indicates better discrimination ability [19]. A c-index of 0.5 represents agreement by chance alone, while a c-index of 1 indicates perfect discrimination. Two-sided *P* values < 0.05 were considered statistically significant. Internal validation of the model was performed using a bootstrap technique with 100 resamples from our original dataset [18].

## 3. Results

### 3.1. Patient Characteristics

The detailed clinical characteristics of the included patients are displayed in Table 1. The patients are divided into five groups (T1 stage, T2 stage, T3 stage, T4 stage, and TX), and different groups are in different columns, and each row represents various baseline information, and the proportion of the number in each predictor variable in the T stage also is shown in the Table 1. Among the 17845 patients collected from the SEER database, the median age at diagnosis was 65.5 years, and 79.5% of patients were male. The distributions of esophageal cancers were 5.0%, 36.3%, 43.6%, and 15.0%. The proportion of N0, N1, N2, N3, and NX was 19.6%, 8.0%, 3.6%, 1.9%, and 66.3% and the proportion of M0, M1, and MX was 69.0%, 30.1%, and 0.9%. The median tumor size was 5.01 cm (interquartile range: 3.0-6.5 cm). The proportion of no surgery, local tumor excision or local tumor excision with destruction, esophagectomy with laryngectomy, and/or gastrectomy was 64.3%, 2.8%, 8.5%, and 24.2%, respectively.

LNE: lymph node examined; SCN: squamous cell neoplasms; ANA: adenomas and adenocarcinomas; EN: epithelial neoplasms; CMSN: cystic, mucinous, and serous neoplasms; CEN: complex epithelial neoplasms; local tumor ED: local tumor excision or/with destruction; esophagectomy LG: esophagectomy with laryngectomy and/or gastrectomy.

### 3.2. Prognostic Value of Tumor Size in Different T Stages

**Figure 1:**
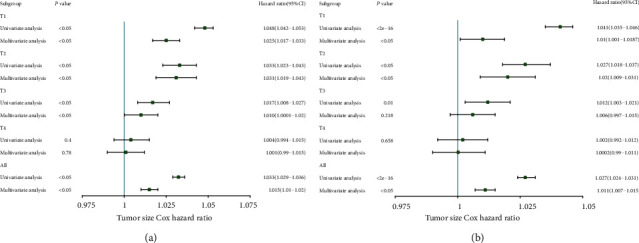
The hazard ratio (HR) of tumor size in predicting survival on esophageal cancer based on the different T stages: (a) CSS set and (b) OS set.

**Figure 2:**
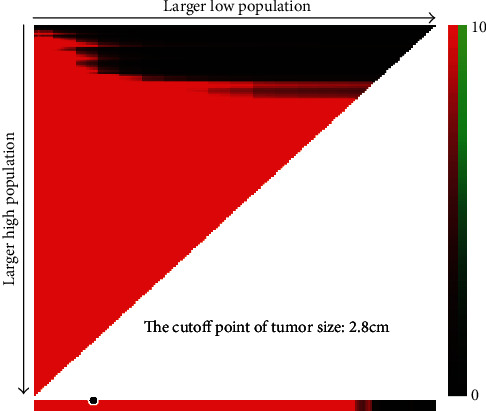
Division of tumor size by the cutoff point produced by the X-Tile plot.

**Figure 3:**
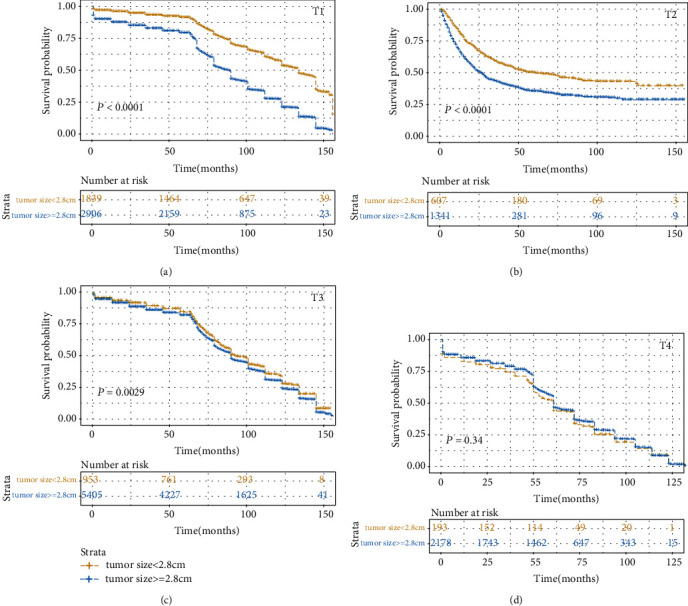
Kaplan-Meier survival curves compared tumor size less than 2.8 cm with tumor greater than or equal to 2.8 cm based on different T stages in the CSS set: (a) in the T1 stage, (b) in the T2 stage, (c) in the T3 stage, and (d) in the T4 stage.

**Figure 4:**
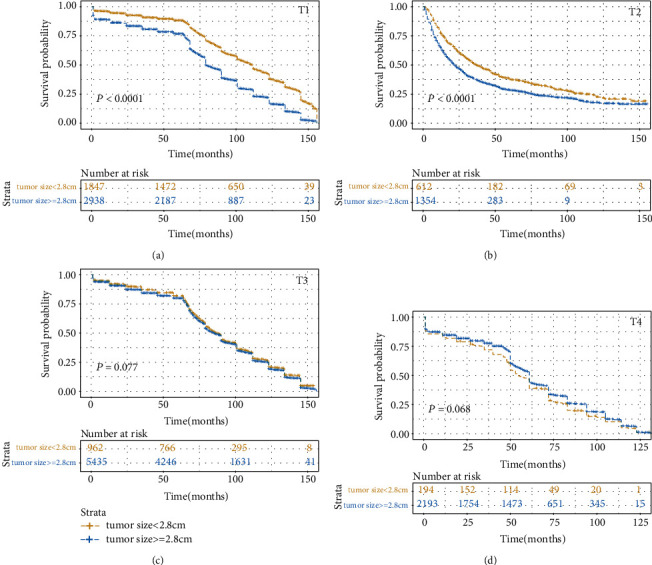
Kaplan-Meier survival curves compared tumor size less than 2.8 cm with tumor greater than or equal to 2.8 cm based on different T stages in the OS set: (a) in the T1 stage, (b) in the T2 stage, (c) in the T3 stage, and (d) in the T4 stage.

The prognostic value of tumor size was evaluated by univariate and multivariate Cox proportional hazards regression analyses (Table 2). Results of the univariate and multivariate analysis are presented in Figure 1. The result found that the T stage could affect the independent prognostic value of tumor size in the CSS or the OS set. In the CSS set, the tumor size in T1, T2, and T3 stage cancers was an independent prognostic factor, and the hazard ratio (HR) (reflected death risk) was the highest in T2, but the HR in T3 stage was nearly 1, which indicated the prognostic significance of tumor size in T3 stage is weaker than those in T1-2 stages. In the OS setting, tumor size was an independent risk factor in T1 and T2 cancer. Interestingly, the risk of death reflected by HR was the highest in T2 in both the OS set and CSS set. We then categorized the tumor size according to the X-Tile (Figure 2) and plot the survival curves according to tumor size within each T stage in order to determine the difference in survival outcome more intuitively (Figures 3 and 4). The results demonstrated that there was a significant difference in survival between patients with tumors < 2.8 cm in the T1 and T2 stages of both the CSS set and OS set; however, the difference in survival outcome was insignificant in T3 and T4 stages.

In summary, the tumor size had a significant influence on death risk in the T1 and T2 stages of esophageal cancer. Furthermore, when the tumor invades the adventitia and adjacent structures, tumor size would not be a prognostic factor in esophageal cancers. Thus, tumor size is a potential prognostic factor in T1-2 stage esophageal cancer.

AOPI: Asian or Pacific Islander; A I/A N: American Indian/Alaska Native; LNE: lymph node examined; SCN: squamous cell neoplasms; ANA: adenomas and adenocarcinomas; EN: epithelial neoplasms; CMSN: cystic, mucinous, and serous neoplasms; CEN: complex epithelial neoplasms; local tumor ED: local tumor excision or/with destruction; esophagectomy LG: esophagectomy with laryngectomy and/or gastrectomy.

### 3.3. Discriminatory Ability of Tumor Size in Different T Stages

The discriminatory ability of tumor size was compared to independent prognostic factors identified by Cox proportional hazards regression analysis by c-index (Table 3). Among all patients, the c-index of tumor size was only less than the N stage and the surgery status and was almost equal to the T stage and M stage in both the OS set and CSS set. Moreover, a subgroup analysis was conducted according to different T stages, and tumor size was found to be a valuable prognostic factor, which outperformed many other widely used prognostic factors in T1-2 stage. In the T1 stage, the c-index of tumor size (CSS set: 0.63, OS set: 0.604) was higher than those in age (CSS set: 0.523, OS set: 0.537), sex (CSS set: 0.509, OS set: 0.510), grade (CSS set: 0.555, OS set: 0.544), N stage (CSS set: 0.598, OS set: 0.585), and M stage (CSS set: 0.592, OS set: 0.568). In the T2 stage, the c-index of tumor size (CSS set: 0.589, OS set: 0.572) was higher than those in age (CSS set: 0.557, OS set: 0.566), sex (CSS set: 0.502, OS set: 0.502), grade (CSS set: 0.539, OS set: 0.544), and M stage (CSS set: 0.572, OS set: 0.561). Moreover, when the T stage increased, the c-index of tumor size decreased. In particular, when the T stage was increased above T2, the predictive ability of tumor size became insignificant.

**Figure 5:**
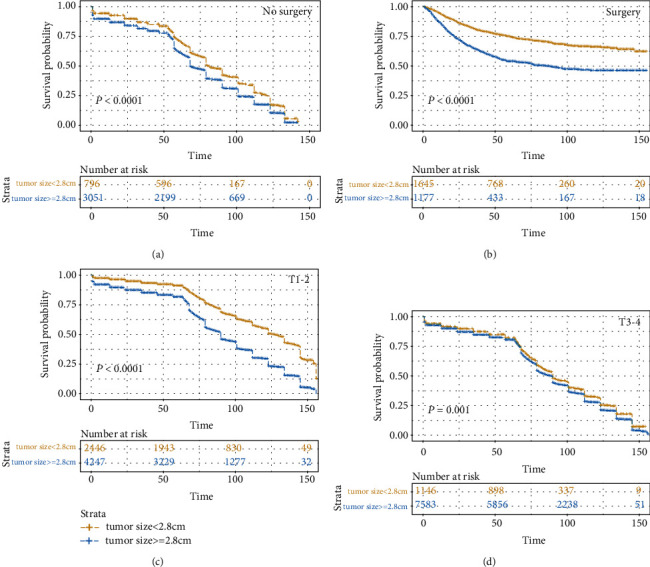
Kaplan-Meier survival curves compared tumor size less than 2.8 cm with tumor size greater than or equal to 2.8 cm: (a) in the no surgery group of the T1-2 stage, (b) in the surgery group of the T1-2 stage, (c) in the T1-2 stage, and (d) in the T3-4 stage.

Further exploration of the value of tumor size in predicting survival is ongoing in T1-2 esophageal cancers. Subgroup analysis was also performed in T1-2 patients based on the N stage (N0, N+) (Table 4). Subgroup analysis in the T1-2 patients was performed based on the therapy (no surgery, surgery) in order to analyze the effects of therapy on the discriminative ability of prognostic factors (Table 5). In addition, we found that the discriminatory ability of tumor size in the N0 group outperformed that of other prognostic factors. In the N+ group, the discriminatory ability of tumor size (CSS set: 0.564, OS set: 0.553) was also higher than those of age (CSS set: 0.517, OS set: 0.531), sex (CSS set: 0.511, OS set: 0.509), grade (CSS set: 0.542, OS set: 0.548), and M stage (CSS set: 0.548, OS set: 0.542). Furthermore, we found that the discriminatory ability of tumor size in the surgery group (CSS set: 0.627, OS set: 0.589) and no surgery group (CSS set: 0.589, OS set: 0.534) outperformed that of other prognostic factors. In addition, we plotted survival curves to show the difference in survival outcome between T1-2 stage and T3-4 stage and between the patients in the surgery group and the no surgery group in order to analyze the influence of the treatment on the result (Figure 5). The result demonstrates a significant difference in survival between T1-2 stage patients with tumors < 2.8 cm and those with tumors ≥ 2.8 cm in both the surgery group and the no surgery group.

In summary, the predictive ability of tumor size reduced as the T stage increased; thus, tumor size was more valuable as a predictor of survival in T1-2 stage esophageal cancer patients. Furthermore, therapy has no significant influence on the dominance for discrimination of tumor size in T1-2 stage patients. Consequently, tumor size should be added in the T staging system.

### 3.4. Construction Nomogram Based on Tumor Size in T1-2 Esophageal Cancers

**Figure 6:**
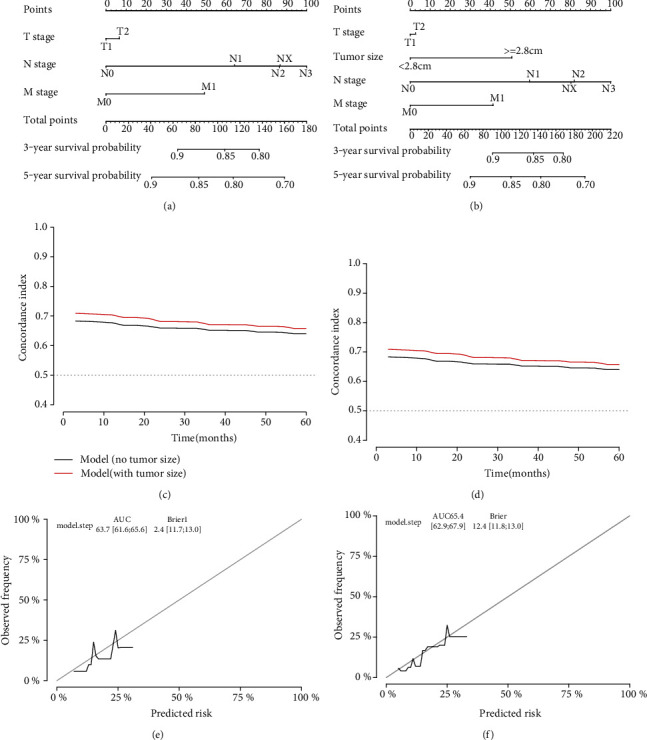
Construction of the nomogram in the T1-2 stage. (a) Nomogram incorporated tumor size and the TNM stage. (b) Nomogram incorporated with the TNM stage. (c) c-index of the nomogram by the times in the CSS set. (d) c-index of the nomogram by the times in the OS set. (e) The internal validation of the model with no tumor size. (f) The internal validation of the model with tumor size.

Nomograms are widely used as prognostic tools in medicine and function to provide references for clinical diagnosis and prognosis [19]. Therefore, to further explore the clinical application of tumor size in the TNM system, we developed a nomogram based on multivariate Cox analysis to explore the significance of adding tumor size to early T stage esophageal cancer. The T stage, N stage, and M stage were incorporated into nomogram 1 (Figure 6(a)), while tumor size, T stage, N stage, and M stage were incorporated in nomogram 2 (Figure 6(b)). The c-index by the different times of the nomogram indicates the model was more accurate when the model incorporated tumor size (Figures 6(c) and 6(d)). Furthermore, the bootstrap-corrected c-statistic of the model with tumor size was still higher than the bootstrap-corrected c-statistic of the model without tumor size (Figures 6(e) and 6(f)).

In summary, the addition of tumor size to the T stage system for T1-2 stage patients should be considered.

## 4. Discussion

The current research explored the relationship between the predictive ability and prognostic value of tumor size and T stage in esophageal cancers. The results demonstrated that an increase in T stage could negatively affect the value of tumor size on prognosis. In the T1-2 stage patients, larger tumor size was related to worse outcomes; however, tumor size was not found to be an independent prognostic factor in the T3-4 stage. Moreover, the predictive ability of tumor size outperformed that of many other widely used prognostic factors. In addition, as the T stage advanced, the discriminatory power of tumor size was dramatically weakened, especially in the T3-4 stage.

The view that tumor size has a negative effect on predicting survival is supported by many investigators; however, the relationship between the T stage and discriminatory value of tumor size is still underappreciated [20–23]. Our study identified tumor size as an independent prognostic factor in T1-2 stage esophageal cancer patients, which is in line with previous results [12, 13]. The most obvious finding to emerge from the current analysis is that the predictive ability of tumor size decreased as the T stage increased.

We consider that there are two possible reasons for the negative influence of the T stage on tumor size. Firstly, when the tumor infiltrates the adventitia (T3 stage), the calculation of the tumor size of esophageal cancers may be inaccurate. Indeed, Sillah et al. [24] highlighted that Computed Tomography (CT) overestimated esophageal tumor length in later stage cancers. In this study, the tumor length by CT was longer than the pathological length with a mean difference of 1.67 cm. Importantly, some studies have shown that the endoscopic esophageal tumor length could be used to independently predict the survival of patients with esophageal cancer; consequently, it has been suggested that the endoscopic tumor length should be included in the staging system of esophageal cancer [22, 25]. Endoscopic tumor length may be useful to calculate the tumor size in esophageal cancer when the esophageal tumor is confined to the submucosa (T1-2 stage), in which the main growth pattern is horizontal growth. However, as the tumor infiltrates over the adventitia (T3-T4 stage), vertical growth is the main growth pattern. Therefore, the significance of tumor size in prognosis is reduced in T3-4 stage cancer. The result of our study supports this viewpoint.

Most interestingly, our result showed that the use of tumor size as a predictor in T1-2 stage esophageal cancer patients outperformed many widely used clinical prognostic factors with regard to predictive ability. The long survival of T1-2 esophageal cancer patients (usually regarded as early-stage) is more dependent on the lymph node status [26]. Some research has demonstrated that tumor size is a predictor of node positivity in esophageal cancer patients who have not received neoadjuvant treatment [27]; this provides powerful evidence to support that the tumor size could be applied in future revisions of the AJCC TNM staging system for T1-2 stage esophageal cancers. Moreover, some studies have found that the tumor size, especially in nodal-negative patients or those with T3-4 grade, is a predictive factor for long-term survival in elderly patients with esophageal cancer; this result appears to be contrary to that of the current study. Therefore, the relationship between T stage and the discriminatory and prognostic value of tumor size based on a different age group of patients should be explored further.

The present study has several limitations. First, the research was limited by the retrospective nature of the study. Second, the study only comprised data from the SEER database and, therefore, lacked validation by external data. Third, the surgical margin status was not available in the SEER database, which may have impacted the result. Fourth, the study did not consider multifocal tumors, which might affect the observer result. Notwithstanding these limitations, the research assessed the influence of T stage on the predictive and prognostic value of tumor size in esophageal cancer for the first time. Importantly, we demonstrate that tumor size has a pivotal effect on the prognosis and predictability in T1-2 stage esophageal cancer patients.

## 5. Conclusion

Our study revealed that the prognostic effect of tumor size in T1-2 stage esophageal cancer is clearly supported. Besides, the ability of tumor size in predicting survival gradually decreased as the T stage increased, while the discriminative ability of tumor size is better than many other clinical prognostic factors in the T1-2 stage. Above all, the results suggest that the tumor size can be considered to be a valuable factor in predicting prognosis in T1-2 esophageal cancer patients.

## Figures and Tables

**Table 1:** Baseline characteristics of esophagus cancer patients by T stage.

	All (%)	T1 (%)	T2 (%)	T3 (%)	T4 (%)	TX (%)
Age (year)	65.6	66.7	65.9	64.8	63.9	67.3
Sex						
Male	14192 (79.5)	3732 (78.7)	1521 (74.3)	5177 (81.4)	1873 (79.0)	1889 (78.0)
Female	3653 (20.5)	1013 (21.3)	527 (25.7)	1181 (18.6)	498 (21.0)	534 (22.0)
Histology						
SCN	5862 (32.8)	1482 (31.2)	601 (29.3)	1898 (29.9)	1018 (42.9)	863 (35.6)
ANA	10442 (58.5)	2889 (60.9)	1223 (59.7)	3896 (61.3)	1112 (46.9)	1322 (54.6)
EN	568 (3.2)	165 (3.5)	30 (1.5)	127 (2.0)	110 (4.6)	136 (5.6)
CMSN	812 (4.6)	180 (3.8)	77 (3.8)	373 (5.9)	108 (4.6)	74 (3.1)
CEN	161 (0.9)	29 (0.6)	17 (0.8)	64 (1.0)	23 (1.0)	28 (1.1)
Grade						
Grade I	898 (5.0)	359 (7.6)	107 (5.2)	257 (4.0)	99 (4.2)	76 (3.1)
Grade II	6481 (36.3)	1832 (38.6)	841 (41.1)	2275 (35.8)	784 (33.1)	749 (30.9)
Grade III-IV	7786 (43.6)	1781 (37.5)	737 (36.0)	3033 (47.7)	1066 (45.0)	1169 (48.2)
Unknown	2680 (15.0)	773 (16.3)	263 (12.8)	793 (12.5)	422 (17.8)	429 (17.7)
N stage						
N0	3491 (19.6)	1181 (24.9)	605 (29.5)	1473 (23.2)	177 (7.5)	54 (2.2)
N1	1416 (8.0)	239 (5.0)	229 (11.2)	808 (12.7)	111 (4.7)	29 (1.2)
N2	645 (3.6)	39 (0.8)	74 (3.6)	474 (7.5)	54 (2.3)	4 (0.2)
N3	346 (1.9)	9 (0.2)	21 (1.0)	269 (4.2)	44 (1.9)	3 (0.1)
NX	11837 (66.3)	3258 (68.7)	1010 (49.3)	3304 (52.0)	1974 (83.3)	2291 (94.6)
M stage						
M0	12321 (69.0)	3498 (73.7)	1686 (82.3)	5103 (80.3)	1207 (50.9)	827 (34.1)
M1	5368 (30.1)	1247 (26.3)	262 (12.8)	1255 (19.7)	1164 (49.1)	1440 (59.4)
MX	156 (0.9)	0 (0.0)	0 (0.0)	0 (0.0)	0 (0.0)	156 (6.4)
Tumor size (cm), median (IQR)	5.01 (3.0-6.5)	4.04 (1.8-5.2)	4.18 (2.2-5.0)	5.16 (3.2-6.5)	6.38 (4.0-8.0)	5.85 (4.0-7.2)
LNE						
0-2	12458 (69.8)	3441 (27.6)	1110 (54.2)	3546 (55.8)	2042 (86.1)	2319 (95.7)
>2	5169 (28.7)	1252 (24.4)	812 (39.6)	2746 (43.2)	304 (12.8)	55 (2.3)
Surgery status						
No surgery	11468 (64.3)	2863 (25.0)	984 (48.0)	3307 (52.0)	2007 (84.6)	2307 (95.2)
Local tumor ED	502 (2.8)	400 (80.0)	28 (1.4)	28 (0.4)	199 (8.4)	27 (1.1)
Esophagectomy	1508 (8.5)	412 (27.3)	262 (12.8)	732 (11.5)	74 (3.1)	28 (1.1)
Esophagectomy LG	4319 (24.2)	1050 (24.3)	670 (32.7)	2283 (35.9)	267 (11.3)	49 (2.0)

**Table 2:** Univariate and multivariate Cox proportional hazards regression analyses in 17845 patients.

CSS	Univariate cox analysis			Multivariate cox analysis		
	HR	SE	P	HR	SE	P
Age	1.004	0.0008	5.54*e*-08^∗∗^	1.004	0.0008	1.74*e*-06^∗∗∗^
Sex						
Male	Reference			Reference		
Female	0.976	0.0228	0.290	0.9646	0.0237	1.12872
Histology						
SCN	Reference			Reference		
ANA	0.915	0.0197	6.27*e*-06^∗∗∗^	1.001	0.0209	0.99621
EN	1.123	0.0506	0.022	0.9837	0.0515	0.75023
CMSN	1.004	0.0436	0.919	1.014	0.0447	0.75682
CEN	1.106	0.0934	0.280	1.041	0.0939	0.67022
Grade						
Grade I	Reference			Reference		
Grade II	1.429	0.0507	<0.05	1.169	0.0097	0.00213
Grades III-IV	1.689	0.0501	<0.05	1.241	0.0508	2.12*e*-05^∗∗∗^
Unknown	1.538	0.0538	<0.05	1.143	0.0543	0.01376
T stage						
T1	Reference			Reference		
T2	0.956	0.0362	0.209	0.976	0.0365	0.50689
T3	1.203	0.0244	<0.05	1.104	0.0253	9.41*e*-05^∗∗∗^
T4	1.613	0.0295	<0.05	1.221	0.0305	5.76*e*-11^∗∗∗^
TX	1.755	0.0291	<0.05	1.244	0.0308	1.42*e*-12^∗∗∗^
N stage						
N0	Reference			Reference		
N1	1.899	0.0431	<0.05	1.667	0.0441	<2*e*-16
N2	2.228	0.0537	<0.05	2.056	0.0552	<2*e*-16
N3	2.714	0.0644	<0.05	2.381	0.0662	<2*e*-16
NX	2.398	0.0293	<0.05	1.420	0.0577	1.25*e*-09^∗∗∗^
M stage						
M0	Reference			Reference		
M1	1.702	0.0187	<0.05	1.306	0.0212	<2*e*-16
MX	1.677	0.0878	3.99e-09	1.178	0.0911	0.0717
Tumor size	1.033	0.0017	<0.05	1.015	0.0023	7.81*e*-11^∗∗∗^
Lymph node examined						
≤2	Reference			Reference		
>2	0.580	0.0223	<0.05	0.9539	0.0657	0.47227
Surgery statue						
No surgery	Reference			Reference		
Local tumor ED	0.272	0.0887	<0.05	0.3537	0.0907	<2*e*-16
Esophagectomy	0.494	0.0395	<0.05	0.67	0.0636	2.95*e*-10^∗∗∗^
Esophagectomy LG	0.555	0.0240	<0.05	0.7234	0.0576	1.84*e*-08^∗∗∗^

**Table 3:** The predictive ability of prognostic factors for CSS and OS in esophagus cancer.

c-index	All	T1	T2	T3	T4
OS					
Tumor size	0.550	0.604	0.572	0.512	0.513
Age	0.522	0.537	0.566	0.514	0.519
Sex	0.500	0.510	0.502	0.509	0.501
Grade	0.524	0.544	0.544	0.506	0.514
T stage	0.549	NA	NA	NA	NA
N stage	0.559	0.585	0.618	0.546	0.524
M stage	0.551	0.568	0.561	0.523	0.526
Surgery	0.565	0.62	0.623	0.528	0.524
CSS					
Tumor size	0.563	0.63	0.589	0.519	0.514
Age	0.513	0.523	0.557	0.507	0.514
Sex	0.502	0.509	0.502	0.51	0.505
Grade	0.531	0.555	0.539	0.509	0.518
T stage	0.561	NA	NA	NA	NA
N stage	0.566	0.598	0.629	0.551	0.529
M stage	0.565	0.592	0.572	0.53	0.532
Surgery	0.574	0.641	0.629	0.532	0.528

**Table 4:** The predictive ability of prognostic factors for CSS and OS in T1-2 stage esophagus cancer patients based on different N stages.

c-index	T1-2N0	T1-2N+
OS		
Tumor size	0.55	0.553
Age	0.544	0.531
Sex	0.509	0.509
Grade	0.533	0.548
M stage	0.51	0.542
Surgery	0.536	0.561
CSS		
Tumor size	0.577	0.564
Age	0.527	0.517
Sex	0.513	0.511
Grade	0.562	0.542
M stage	0.517	0.548
Surgery	0.549	0.559

**Table 5:** The predictive ability of prognostic factors for CSS and OS in T1-2 stage esophagus cancer patients based on different therapy situations.

c-index	No surgery	Surgery
OS		
Tumor size	0.534	0.589
Age	0.509	0.555
Sex	0.504	0.499
Grade	0.516	0.558
M stage	0.527	0.517
N stage	0.505	0.575
CSS		
Tumor size	0.549	0.627
Age	0.503	0.521
Sex	0.507	0.506
Grade	0.519	0.582
M stage	0.545	0.526
N stage	0.505	0.594

## Data Availability

The data from Surveillance, Epidemiology, and End Results (SEER) database was analyzed in the study.
